# Kernel Causality Among Teacher Self-Efficacy, Job Satisfaction, School Climate, and Workplace Well-Being and Stress in TALIS

**DOI:** 10.3389/fpsyg.2021.694961

**Published:** 2021-08-18

**Authors:** Xue Zhang, Chunyang Zhao, Yuqiao Xu, Shanhuai Liu, Zhihui Wu

**Affiliations:** ^1^China Institute of Rural Education Development, Northeast Normal University, Changchun, China; ^2^School of Mathematics and Statistics, Northeast Normal University, Changchun, China; ^3^Faculty of Education, Northeast Normal University, Changchun, China

**Keywords:** kernel causality, generalized measure of correlation, nominal response model, extreme response style, TALIS

## Abstract

Teachers play an important role in the educational system. Teacher self-efficacy, job satisfaction, school climate, and workplace well-being and stress are four individual characteristics shown to be associated with tendency to turnover. In this article, data from the Teaching and Learning International Survey (TALIS) 2018 teacher questionnaire are analyzed, with the goal to understand the interplay amongst these four individual characteristics. The main purposes of this study are to (1) measure extreme response style for each scale using unidimensional nominal response models, and (2) investigate the kernel causal paths among teacher self-efficacy, job satisfaction, school climate, and workplace well-being and stress in the TALIS-PISA linked countries/economies. Our findings support the existence of extreme response style, the rational non-normal distribution assumption of latent traits, and the feasibility of kernel causal inference in the educational sector. Results of the present study inform the development of future correlational research and policy making in education.

## Introduction

In recent years, teacher turnover has become an increasingly prominent issue surrounding the topic of teaching quality. It was found that a high sense of teacher self-efficacy has an indirect effect on later job satisfaction via the mediational role of engagement (Granziera and Perera, 2019), controls professional stress (Bangs and Frost, 2012), increases teacher well-being (Smylie, 1988), and reduces quitting intentions (Tschannen-Moran and Hoy, 2001; Wang et al., 2015). Job satisfaction is considered as a critical predictor of teacher recruitment and retention (e.g., Renzulli et al., 2011; Wang et al., 2015), Moreover, job satisfaction is also linked to teachers’ occupational well-being and motivation, and some subscales are related to the disciplinary climate (Dicke et al., 2020). Teachers’ perceptions of the school climate have been found to be a key predictor of teacher self-efficacy, job satisfaction, and sense of stress (e.g., Borg, 1990; Hoy and Woolfolk, 1993; Kim and Loadman, 1994; Collie et al., 2012; Wilson et al., 2020). Warr (1999) and Harter et al. (2002) found that a high sense of workplace well-being can contribute to job retention.

Björnsson (2020) assessed the variations in Nordic teachers’ self-efficacy in multicultural classrooms using the Teaching and Learning International Survey (TALIS) 2018 data, in which the influences of workload stress, teacher-student relations, job satisfaction, and disciplinary climate were also considered. The TALIS, which were organized by the Organization for Economic Co-operation and Development (OECD) from 2008 to 2018, assessed measures such as working conditions, beliefs, and attitudes from principals and teachers, with the goal to help countries review and develop policies that promote conditions for effective teaching and learning (Ainley and Carstens, 2018). Hereafter, response data from the third round of TALIS (i.e., 2018) are used.

Although research is emerging on teacher self-efficacy (TSE), job satisfaction (JS), school climate (SC), or workplace well-being and stress (WWS), it is critical to understand the internal relationship amongst them. However, the causality has not been taken into account. The primary questions of this article are: (1) to what extent do the extreme response styles vary across different countries/economies and (2) what variation is evident in causalities amongst TSE, JS, SC, and WWS across countries/economies? We propose the following hypotheses: H1. There exist extreme response styles for these four dimensions in TALIS 2018 data; H2. TSE, JS, and SC are positively correlated with each other, and negatively correlated with WWS; and H3. Distinct kernel causal paths exist across different countries/economics.

## Background

### Teacher Self-Efficacy (TSE)

Self-efficacy was first defined by Bandura (1977) as belief in one’s capability to accomplish desired outcomes, which can be grounded in mastery experience, vicarious experiences, social persuasion, and physical and emotional states. Similarly, TSE is defined as teachers’ beliefs in their ability to solve challenges and difficulties accumulated within a teacher’s professional career (Armor et al., 1976; Bandura, 1997; Tschannen-Moran and Hoy, 2001; Schwarzer and Hallum, 2008). Three dimensions of TSE are operationalized by the TALIS 2018 team (OECD, 2019, p. 285): self-efficacy in classroom management (SECLS), self-efficacy in instruction (SEINS), and self-efficacy in student engagement (SEENG).

### Job Satisfaction (JS)

Job satisfaction can be defined as “*a pleasurable or positive emotional state resulting from the appraisal of one’s job or job experiences*” by Locke(1976, p. 1300), “*the extent to which people like (satisfaction) or dislike (dissatisfaction) their jobs*” by Spector(1997, p. 2), or “*the state of mind determined by the extent to which the individual perceives her/his job-related needs to be being met*” by Evans(1997, p. 833). Wang et al. (2018) conducted collaborative practices on teacher JS to examine how teachers perceive the comparison of actual job outcomes with desired ones.

In the TALIS 2018, three dimensions of JS are conceptualized and measured (OECD, 2019, p. 302): teacher JS with work environment (JSENV), job satisfaction with profession (JSPRO), and satisfaction with target class autonomy (SATAT). The JSENV scale assesses the satisfaction of working at a specific school, the JSPRO scale focuses on a global evaluation of the decision to become a teacher, and the SATAT scale measures the self-report of teaching at a specific class.

### School Climate (SC)

The definition of SC is still an open question. SC can be referred to as the quality and character of school life depending on patterns of one’s personal experience (Cohen et al., 2009, p. 10). Zullig et al. (2010) found that social relationships in the school climate scale could be subdivided into three distinct areas: *overall social environment*, *positive student–teacher relationships*, *and perceived exclusion/privilege*. Among these areas, positive student-teacher relationships correlated positively with other school climate domains, and perceived exclusion/privilege correlated negatively with school connectedness (Zullig et al., 2010). Interested readers can refer to Zullig et al. (2010) and Thapa et al. (2013) for more school climate domains that are historically common.

The SC consisted of three subscales in the TALIS 2018 (OECD, 2019, p. 334): teachers’ perceived disciplinary climate (DISC), teacher-student relations (STUD), and participation among stakeholders and teachers (STAKE). Among them, the DISC scale evaluates the class discipline, the STUD scale examines the self-report of the relationship between teachers and students, and the STAKE scale measures the distributed leadership.

### Workplace Well-Being and Stress (WWS)

As a comprehensive social, physical, and emotional sense (Warr, 1990), employees’ well-being not only matters to health and duty of care, but also links tangibly with effectiveness in the workplace (Lévi, 2000). In addition, workplace well-being can be treated as a fundamental element of successful organizations. Lazarus (1966) found that “*stress arises when individuals perceive that they cannot adequately cope with the demands being made on them or with threats to their well-being*.” Further, workplace stress has been defined by Colligan and Higgins (2006) as the variation of physical or/and mental response to an appraised challenge or threat posed by the workplace.

The WWS scale involved three subscales in the TALIS 2018 (OECD, 2019, p. 319): workplace well-being and stress (WELS), workload stress (WLOAD), and student behavior stress (STBEH). The WELS scale measures workplace well-being, stress, and its effect on other things; the WLOAD scale evaluate the stress connected to workload, and the STBEH scale evaluate the stress connected to classroom and student management.

### Response Styles

Under the survey component of large-scale assessment, psychological constructs (e.g., beliefs and attitudes) are measured by rating or Likert-type scale self-reports. Baumgartner and Steenkamp (2001) found that items in such assessments are vulnerable to response styles (RS), or differences in how respondents tend to use the response options. Frequently used RS consist of extreme response style (ERS), midpoint response style (MRS), and acquiescent response style (ARS), which mean a tendency to choose extreme response options, to excessively use the midpoint, and to agree with the item, respectively. In international studies, different response styles may be because of cultural variabilities (e.g., Hui and Triandis, 1989). For instance, Buckley (2009) considered heterogeneous response scales across countries for PISA 2006 data. Ju and Falk (2019) used a multilevel multidimensional nominal response model to measure ERS using TALIS 2013 data.

As recommended by Ju and Falk (2019), the nominal response model (NRM; Bock, 1972) is applied to multi-group analysis to accommodate extreme response styles. Let *y*_ijg_ denote the polytomous scored response of an examinee *j* (*j* = 1, …, *J*) from group *g* (*g* = 1, …, *G*) on item *i* (*i* = 1, …, *I*). The probability of endorsing category *c* (*c* = 1, …, *C*) is given by

(1) $$P\left(y_{\text{ijg}}=c|\theta_{\text{jg}},a_{\text{ig}},b_{\text{icg}}\right)=\frac{\exp\left(a_{\text{ig}}\,\theta_{\text{jg}}+b_{\text{icg}}\right)}{\sum_{k=1}^{C}\exp\left(a_{\text{ig}}\,\theta_{\text{jg}}+b_{\text{ikg}}\right)}$$

where *θ_jg_* denotes the latent trait (e.g., self-efficacy) of examinee *j* from group *g*, *a*_ig_ is the discrimination parameter (i.e., slope) of item *i* for group *g*, and *b*_*icg*_ is the intercept parameter of item *i* on category *c* for group *g*. Moreover, many latent traits, such as self-efficacy (Woods, 2007b) and anxiety (Woods, 2006; Woods and Thissen, 2006), are not typically normally distributed in a population (Woods, 2015). Unsurprisingly, ERS may lead to non-normality of latent traits. Biased estimates of IRT model parameters will be obtained when the normal distribution assumption is violated (Woods, 2015). To handle the non-normal latent trait (LT) distribution, an empirical histogram (EH) method was proposed by Woods (2007a). The EH method is embedded in EM algorithm as a non-parametric approximation of the LT distribution that is simple to implement.

### Kernel Causality^1^

As pointed out by Zheng et al. (2012), Pearson’s correlation coefficient does not apply to asymmetric and/or nonlinear dependence, they proposed the generalized measures of correlation (GMC) to quantify the level of asymmetry in explaining variances. A pair of GMC can be expressed as

(2) $$\left\{\text{GMC}\,\left(X|Y\right),\text{GMC}\,\left(Y|X\right)\right\}=\left\{1-\frac{E\,{\left(\left\{Y-E\,\left(Y|X\right)\right\}\right)}^{2}}{V\,a\,r\,\left(Y\right)},1-\frac{E\,{\left(\left\{X-E\,\left(X|Y\right)\right\}\right)}^{2}}{V\,a\,r\,\left(X\right)}\right\},$$

where *X* and *Y* denote two variables, respectively.

A more refined version of the concept of Granger causality is kernel causality (Vinod, 2017), which can be treated as preliminary determination of causal directions among a set of variables. Kernel causality can be measured by GMC (Zheng et al., 2012). Let *δ* = GMC(*X* | *Y*) – GMC(*Y* | *X*), then kernel cause is defined as follows (Vinod, 2017):

if *δ* < 0, i.e., GMC(*Y* | *X*) > GMC(*X* | *Y*), kernel cause = *X*;

if *δ* = 0, i.e., GMC(*Y* | *X*) = GMC(*X* | *Y*), indeterminate cause;

if *δ* > 0, i.e., GMC(*Y* | *X*) < GMC(*X* | *Y*), kernel cause = *Y*.

The hypothesis is H_0_: *δ* = 0 against H_1_: *δ* ≠ 0, rejecting the null hypothesis (i.e., H_0_) suggests the existence of a statistically significant kernel causality. Vinod (2017) defined P(cause), which was calculated using the maximum entropy bootstrap algorithm, as the larger of the two rejection probabilities (i.e., reject *δ* > 0 and *δ* < 0) in bootstrap resamples. A larger P(cause) means a larger rejection probability of H_0_. When the relation between variables is linear and/or their joint distribution is close to normal, *δ* is close to 0. In addition, 0.7 is recommended as the cut-off point to indicate a plausible kernel causality (Vinod, 2017).

## Materials and Methods

### Data Source and Sample

In this article, we analyzed the response data of teachers in lower secondary schools (ISCED 2) from countries/economies which adopted the TALIS-PISA link option [i.e., Australia, Ciudad Autónoma de Buenos Aires (CABA) – Argentina, Colombia, Czechia, Denmark, Georgia, Malta, Turkey and Vietnam]. We used listwise deletion for missing data. In total, the current study used data collected from 18,571 teachers at 1,512 schools (after initial data cleaning). The average length of teaching experience is 15.99 years with a 10.612 standard deviation, with 66.4% female teachers. Table 1 summarizes sample configuration by country/economy, along with the country code used throughout the article.

**TABLE 1:** Overview of the selected samples.

Country/Economy	Code	#Schools	#Teachers
Australia	AUS	233	2,614
CABA – Argentina	ABA	130	1,653
Colombia	COL	154	2,049
Czechia	CZE	219	3,028
Denmark	DNK	142	1,612
Georgia	GEO	191	1,704
Malta	MLT	55	1,048
Turkey	TUR	196	3,030
Vietnam	VNM	192	1,832
**Total**	–	1,512	18,570

**Source*: OECD, TALIS 2018 Database.*

### Measures

Teacher self-efficacy and WWS are measured by questionnaires tailored to a 4-point Likert-type scale from “*not at all*” (1) to “*a lot*” (4), and the questionnaires of both JS and SC are tailored from “*strongly disagree*” (1) to “*strongly agree*” (4). The sub-test lengths are 12, 13, 13, and 12 for TSE, JS, SC, and WWS, respectively. As a result, the corresponding total scores are 48, 52, 52, and 48 for each scale, respectively. Detailed information of items used in this study is presented in Appendix Table 1.

**FIGURE 1:**
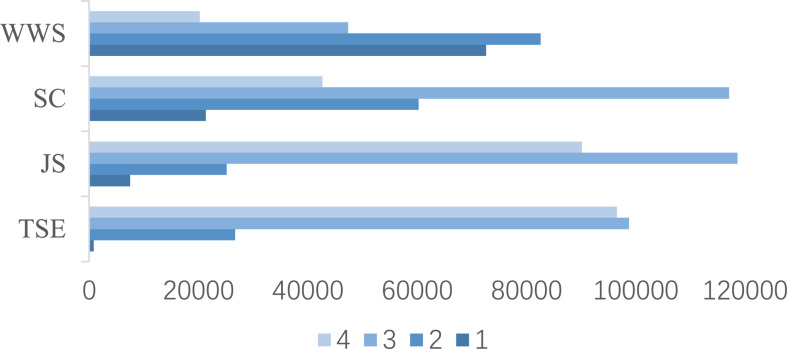
Clustered bar of responses to each scale for total samples.

Responses on each scale are summarized in Figure 1; the darker the color is, the smaller value of the response option is. For TSE, JS, and WWS scales, more than 33% teachers chose extreme options. There were 2,072, 484, and 3 teachers answering the last options of all items for TSE, JS, and SC, respectively. For WWS, which can be treated as a negative sense, 133 teachers chose the first options of all items.

### Analytic Procedure

The analytic procedure consists of two main stages. The first stage is to evaluate multi-group analysis to fit extreme response styles using item scores, and the second stage is to assess kernel causal inference using total scores.

#### Phase 1: Multi-Group Analysis

A multi-group analysis using the unidimensional nominal response model is conducted to fit response data with ERS. R package “mirt” (Chalmers, 2012) is used to fit the TALIS 2018 data. The EM algorithm with empirical histogram (Woods, 2007a) is used to estimate item parameters when latent trait distribution is non-normal or unknown, and the expected a posterior (EAP) method is used to estimate latent trait.

According to the TALIS 2018 technical report (OECD, 2019), each scale of the focused samples reached the metric invariance level, except STBEH subscale, which reached the configural invariance level. Therefore, the invariance = c(‘slopes’) option is applied to support metric-level invariance and keep mean and variance of the population distribution consistent (i.e., mean = 0, variance = 1) for each group. Meanwhile, reliability testing is checked by Cronbach’s alpha reliability coefficient.

#### Phase 2: Kernel Casual Inference

R package “generalCorr” (Vinod, 2020) is used to calculate GMC and evaluate the direction of the kernel causal paths^3^ among TSE, SC, JS, and WWS. Total scores of each scale are used in this phase.

## Results

### Multiple-Group Analysis

Table 2 shows the multi-group analysis results for the nine countries/economies, including Cronbach’s alpha reliability coefficient (*α*), the mean, standard deviation, skewness and excess kurtosis of total scores (i.e., *μ*, *σ*, *β_s_* and *β_k_*)^4^, the average and standard deviation of estimated latent traits (i.e., $\bar{\theta }$ and σ_θ_), and the average and confidence interval of estimated standard errors of latent traits (i.e., SE and CI).

**TABLE 2:** Reliability index, descriptive statistics of total scores, and latent traits.

Code	Scale	α	μ	σ	β_s_	β_k_	${}_{\bar{\theta }}$	σ_θ_	SE	CI
AUS	TSE	0.885	38.85	5.436	–0.179	–0.419	0.3274	0.8781	0.2726	(0.2664, 0.2788)
	JS	0.842	41.48	5.670	–0.403	0.243	0.2828	1.1939	0.3620	(0.3504, 0.3737)
	SC	**0.682**	35.73	4.186	–0.152	0.802	0.1603	1.0180	0.3181	(0.3112, 0.3250)
	WWS	0.839	26.80	6.443	0.366	–0.199	–0.5523	0.9283	0.3318	(0.3242, 0.3393)
ABA	TSE	0.869	40.15	4.798	–0.222	–0.560	0.4045	0.7925	0.2449	(0.2343, 0.2556)
	JS	0.818	43.85	5.022	–0.537	0.323	0.3870	0.6754	0.1558	(0.1405, 0.1711)
	SC	0.724	35.86	4.500	–0.159	0.478	–0.0933	0.5315	0.1883	(0.1842, 0.1924)
	WWS	0.834	21.58	5.768	0.904	0.863	–0.9269	0.8795	0.3660	(0.3542, 0.3778)
COL	TSE	0.885	44.39	4.071	–1.415	2.452	1.1738	0.9031	0.3825	(0.3713, 0.3936)
	JS	0.825	44.33	5.239	–0.743	1.178	0.5786	0.5640	0.2137	(0.2003, 0.2271)
	SC	0.722	36.31	4.431	–0.196	0.994	0.3575	1.2724	0.3312	(0.3182, 0.3441)
	WWS	0.885	27.52	7.535	–0.071	–0.571	–0.5805	1.0579	0.3175	(0.3095, 0.3256)
CZE	TSE	0.848	35.54	4.902	0.073	0.218	–0.0002	0.7560	0.2814	(0.2784, 0.2843)
	JS	0.817	41.28	4.701	–0.169	0.144	–0.0855	0.8376	0.2979	(0.2919, 0.3039)
	SC	0.724	35.18	3.786	–0.038	1.097	–0.2322	0.6414	0.2141	(0.2088, 0.2195)
	WWS	0.824	24.64	5.766	0.367	0.050	–0.6799	0.8424	0.3255	(0.3199, 0.3312)
DNK	TSE	0.859	40.90	4.424	–0.559	0.937	0.6866	0.9946	0.3405	(0.3332,0.3477)
	JS	0.849	42.70	5.539	–0.408	–0.103	0.3590	0.8071	0.2778	(0.2668, 0.2889)
	SC	**0.666**	35.39	3.892	–0.099	0.637	0.2684	0.8671	0.3085	(0.2998, 0.3171)
	WWS	0.848	27.04	6.467	–0.150	–0.311	–0.5134	1.0022	0.2935	(0.2857, 0.3013)
GEO	TSE	0.912	40.11	5.794	–0.491	–0.061	0.5023	1.0478	0.2813	(0.2714, 0.2912)
	JS	0.817	42.21	4.714	0.017	–0.086	0.1827	0.6281	0.1522	(0.1395, 0.1649)
	SC	0.753	36.78	3.798	0.022	0.622	0.1648	1.7042	0.3592	(0.3347, 0.3837)
	WWS	0.820	18.48	4.527	1.059	1.205	–1.2589	0.8655	0.3200	(0.3134, 0.3266)
MLT	TSE	0.886	39.22	5.581	–0.155	–0.643	0.5409	1.0121	0.2567	(0.2419,0.2715)
	JS	0.795	39.44	5.333	–0.176	–0.167	–0.0346	0.8562	0.3142	(0.3041, 0.3243)
	SC	**0.692**	36.22	4.049	–0.035	0.205	–0.2242	0.7028	0.1794	(0.1673, 0.1915)
	WWS	0.829	28.36	6.508	0.197	–0.329	–0.3557	0.7739	0.2843	(0.2760, 0.2927)
TUR	TSE	0.918	39.37	5.747	–0.089	–0.625	0.4147	1.2679	0.2590	(0.2511, 0.2668)
	JS	0.827	39.75	5.969	–0.299	0.151	–0.0338	1.0466	0.3055	(0.2928, 0.3183)
	SC	0.755	35.83	4.699	–0.149	0.856	0.0060	1.3927	0.3080	(0.2976, 0.3184)
	WWS	0.840	24.17	6.002	0.533	0.352	–0.7536	1.0176	0.3726	(0.3658, 0.3794)
VNM	TSE	0.878	41.21	4.714	–0.350	–0.448	0.5086	0.5705	0.1786	(0.1756, 0.1817)
	JS	0.794	40.94	4.242	0.198	0.170	0.0732	0.6063	0.1965	(0.1868, 0.2063)
	SC	**0.624**	34.97	3.125	0.394	1.387	–1.6203	2.4952	0.7907	(0.7495, 0.8318)
	WWS	0.815	25.18	5.463	0.201	–0.319	–0.7180	1.0233	0.3151	(0.3110, 0.3193)

*Boldface entries are the values of Cronbach’s alpha smaller than 0.7.*

All values of Cronbach’s alpha are larger than 0.6, which means an acceptable internal consistency. The Cronbach’s alpha of SC for each country/economy is the smallest, and some are less than an alternative acceptable cut-off point (i.e., 0.7). It appears that results from these data are reliable. The means and standard deviations of each scale’s total scores for each country/economy have similar trends to the case of total samples (shown in Figure 1). TSE of Colombia, JS and SC of Vietnam, and WWS of Georgia are the most centralized around their means.

The values of skewness of total samples are −0.291, −0.328, −0.061, and 0.363 for TSE, JS, SC, and WWS, respectively. And the values of excess kurtosis of total samples are −0.467, 0.224, 0.904, and −0.265 for TSE, SC, JS, and WWS, respectively. In other words, regarding total samples, TSE, JS, and SC are negatively skewed, and WWS is positively skewed. The distributions of TSE and WWS are platykurtic, and the distributions of JS and SC are leptokurtic. The skewness of Australia, CABA - Argentina, Malta, and Turkey are consistent with total samples, and the kurtosis of Australia and Vietnam are consistent with total samples.

**FIGURE 2:**
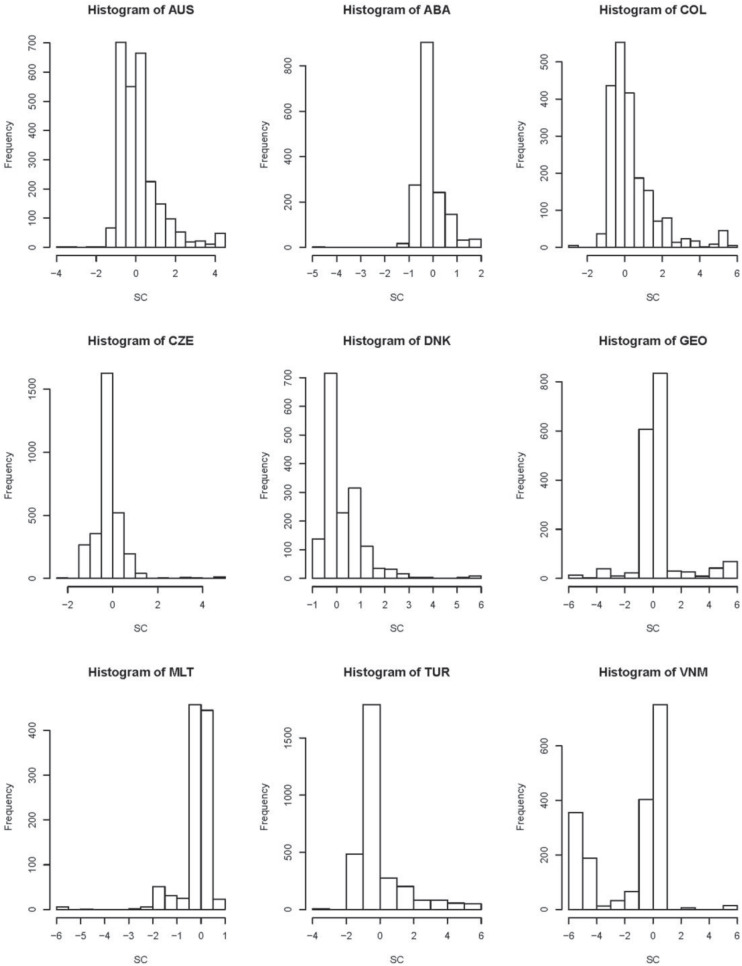
Histogram plots of latent traits (LTs) measured school climate (SC).

In terms of LTs’ estimates (Table 2), Colombia’s teachers have the highest average level of TSE, JS, and SC, Georgia’s teachers have the highest average level of WWS, teachers with the lowest average level of TSE and JS are from the Czechia, teachers with the lowest average level of SC are from Vietnam, and those with the lowest average of WWS are from Malta. TSE estimates of Vietnam, JS estimates of Colombia, SC estimates of CABA – Argentina, and WWS estimates of Malta are the most centralized around the averages of their estimates. On the other hand, the standard errors of TSE for Vietnam, JS for Georgia, and SC and WWS for Malta are smallest. Note that the information of LTs’ estimates of SC for Vietnam presented in Table 2 is consistent as that shown in Figure 2.

**FIGURE 3:**
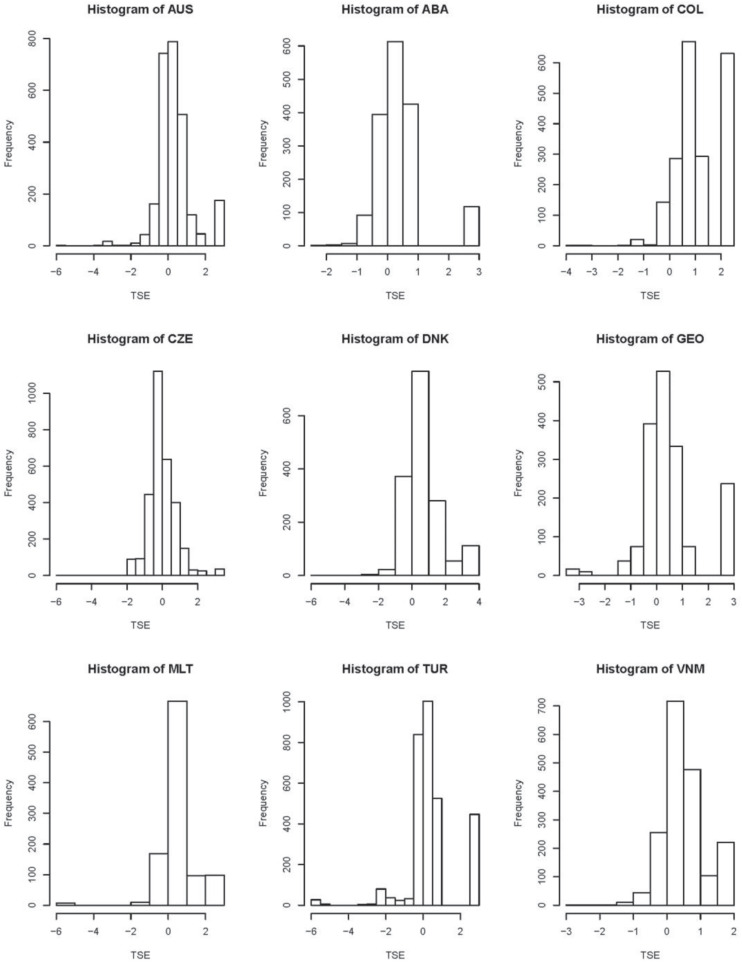
Histogram plots of latent traits (LTs) measured teacher self-efficacy (TSE).

**FIGURE 4:**
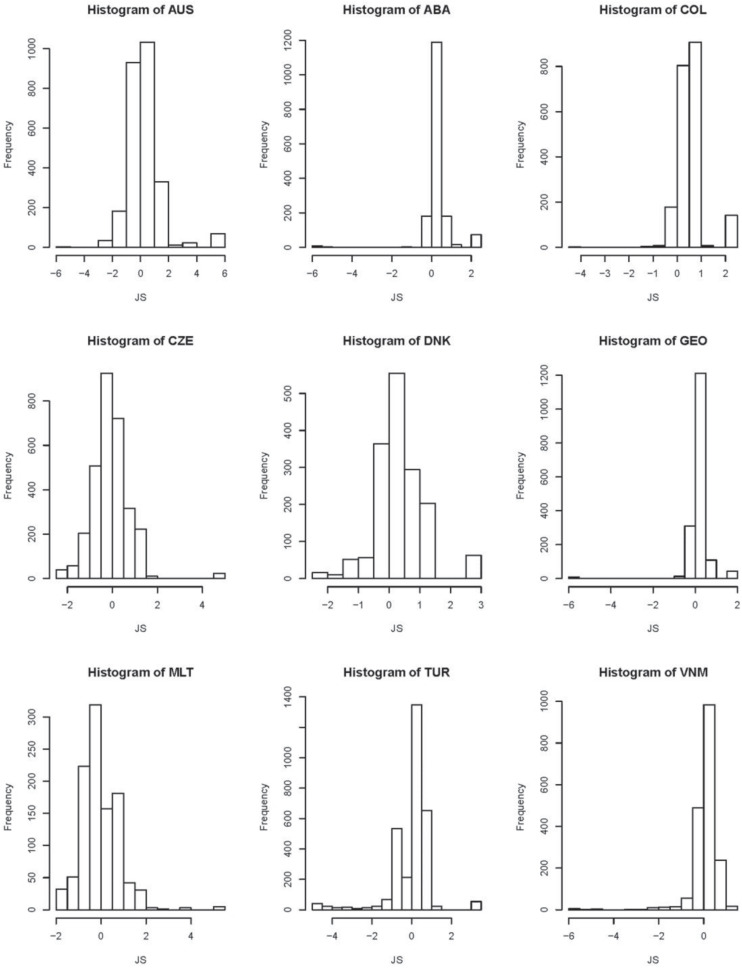
Histogram plots of latent traits (LTs) measured job satisfaction (JS).

**FIGURE 5:**
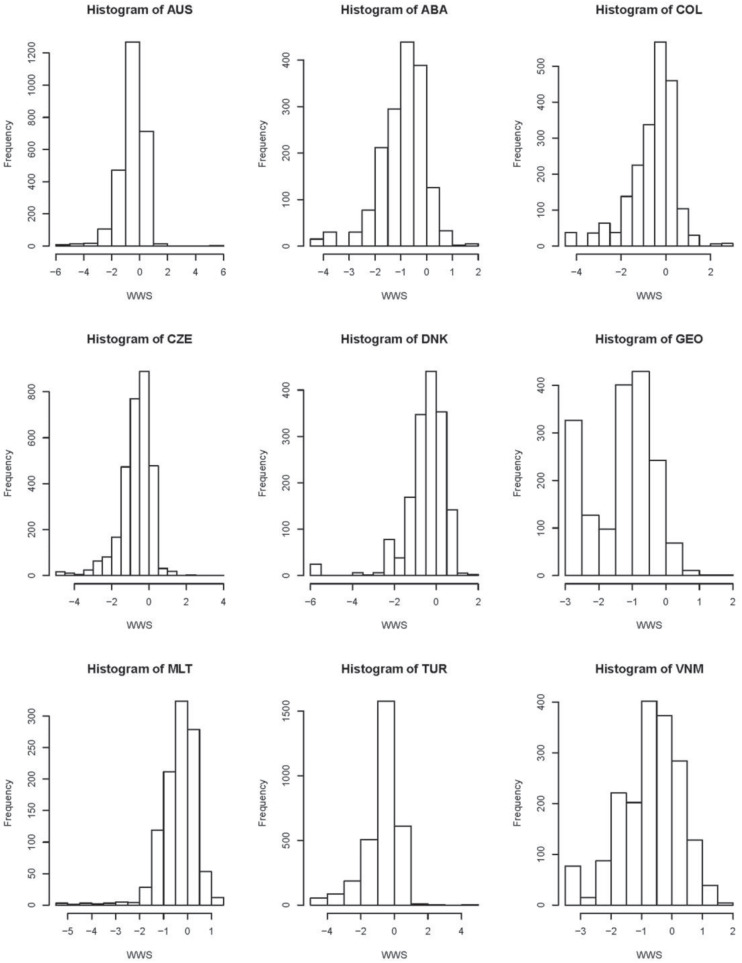
Histogram plots of latent traits (LTs) measured workplace well-being and stress (WWS).

Furthermore, histograms of latent traits measured each scale, which can be considered as an additional information to LT estimates, are shown in Figures 2–5. Most histograms for TSE, JS, and SC are asymmetric. This observation indicates that non-normal assumption is reasonable and necessary.

For TSE, Australia, the Czechia, Denmark, Malta, and Turkey have a wide range of estimates of LTs. For JS and WWS, Australia has the widest range of LTs’ estimates. For SC, Georgia and Vietnam have a wide range of estimates.

### Kernel Causal Inference

Tables 3–11 present the kernel causality measured by GMC and the Pearson’s correlation coefficients among these four scales for different countries/economies, respectively. The absolute values of GMC were larger than the corresponding Pearson’s correlation coefficient, and the trends of these statistics are similar. WWS is negatively correlated with other factors (i.e., TSE, JS, and SC) in most cases, excluding CABA – Argentina, the Czechia and Malta. For Australia, the Czechia, and Denmark, TSE and SC are also negatively correlated with a small correlation coefficient. The largest two correlation coefficients are that between JS and WWS and that between TSE and JS, and the smallest two correlation coefficients are that between TSE and SC and that between SC and WWS in most cases.

**TABLE 3:** Summary of GMC for Australia.

Code	Scenario		*ρ*	GMC(X| Y)	GMC(Y| X)	*δ*	P(cau)	Kernel cause
	X	Y						
AUS	TSE	JS	0.2760	0.2816	0.2950	–0.0134	1	TSE
		SC	–0.0512	–0.0677	–0.0834	0.0157	0.88	SC
		WWS	–0.0963	–0.1080	–0.1477	0.0397	1	WWS
	JS	SC	0.1816	0.1922	0.2008	–0.0086	0.96	JS
		WWS	–0.4871	–0.4930	–0.4922	–0.0008	0.88	JS
	SC	WWS	–0.0664	–0.1275	–0.0810	–0.0465	1	SC

*P(cau), P(cause).*

**TABLE 4:** Summary of GMC for CABA – Argentina.

Code	Scenario		*ρ*	GMC(X| Y)	GMC(Y| X)	*δ*	P(cau)	Kernel cause
	X	Y						
ABA	TSE	JS	0.3255	0.3358	0.3457	–0.0099	0.88	TSE
		SC	0.0546	0.0669	0.0895	–0.0226	0.96	TSE
		WWS	–0.1143	–0.1366	–0.1470	0.0104	0.92	WWS
	JS	SC	0.1811	0.1925	0.1887	0.0038	0.78	SC
		WWS	–0.3273	–0.3415	–0.3427	0.0012	**0.64**	WWS
	SC	WWS	0.0836	0.1388	0.1011	0.0377	0.98	WWS

*P(cau), P(cause). Boldface entries are the values of P(cause) smaller than 0.7.*

**TABLE 5:** Summary of GMC for Colombia.

Code	Scenario		*ρ*	GMC(X| Y)	GMC(Y| X)	*δ*	P(cau)	Kernel cause
	X	Y						
COL	TSE	JS	0.3301	0.3279	0.3487	–0.0208	0.98	TSE
		SC	0.0974	0.1600	0.1657	–0.0057	0.82	TSE
		WWS	–0.2303	–0.2509	–0.2343	–0.0166	1	TSE
	JS	SC	0.2013	0.2606	0.2485	0.0121	0.88	SC
		WWS	–0.3778	–0.3972	–0.3936	–0.0036	0.96	JS
	SC	WWS	–0.0792	–0.0989	–0.1192	0.0203	**0.64**	WWS

*P(cau), P(cause). Boldface entries are the values of P(cause) smaller than 0.7.*

**TABLE 6:** Summary of GMC for the Czechia.

Code	Scenario		*ρ*	GMC(X| Y)	GMC(Y| X)	*δ*	P(cau)	Kernel cause
	X	Y						
CZE	TSE	JS	0.2829	0.2989	0.2847	0.0142	1	JS
		SC	–0.0690	–0.1127	–0.1321	0.0194	**0.66**	SC
		WWS	–0.1775	–0.1887	–0.2067	0.018	0.82	WWS
	JS	SC	0.1560	0.1764	0.1961	–0.0197	0.82	JS
		WWS	–0.4023	–0.4174	–0.4064	–0.0110	1	JS
	SC	WWS	0.0253	0.1295	0.0268	0.1027	1	WWS

*P(cau), P(cause). Boldface entries are the values of P(cause) smaller than 0.7.*

**TABLE 7:** Summary of GMC for Denmark.

Code	Scenario		*ρ*	GMC(X| Y)	GMC(Y| X)	*δ*	P(cau)	Kernel cause
	X	Y						
DNK	TSE	JS	0.2293	0.2535	0.2894	–0.0359	**0.6**	TSE
		SC	–0.0314	–0.0385	–0.0993	0.0608	0.94	SC
		WWS	–0.1437	–0.1650	–0.2040	0.0390	0.98	WWS
	JS	SC	0.2021	0.2296	0.2327	–0.0031	0.82	JS
		WWS	–0.5192	–0.5293	–0.5252	–0.0041	0.76	JS
	SC	WWS	–0.0545	–0.0895	–0.0613	–0.0282	**0.66**	SC

*P(cau), P(cause). Boldface entries are the values of P(cause) smaller than 0.7.*

**TABLE 8:** Summary of GMC for Georgia.

Code	Scenario		*ρ*	GMC(X| Y)	GMC(Y| X)	*δ*	P(cau)	Kernel cause
	X	Y						
GEO	TSE	JS	0.4267	0.4299	0.4333	–0.0034	0.76	TSE
		SC	0.1965	0.2147	0.2568	–0.0455	0.96	TSE
		WWS	–0.1483	–0.1753	–0.1729	–0.0024	**0.66**	TSE
	JS	SC	0.3371	0.3468	0.4105	–0.0637	1	JS
		WWS	–0.2893	–0.3100	–0.3134	0.0034	**0.52**	WWS
	SC	WWS	–0.1101	–0.1675	–0.1884	0.0209	**0.66**	WWS

*P(cau), P(cause). Boldface entries are the values of P(cause) smaller than 0.7.*

**TABLE 9:** Summary of GMC for Malta.

Code	Scenario		*ρ*	GMC(X| Y)	GMC(Y| X)	*δ*	P(cau)	Kernel cause
	X	Y						
MLT	TSE	JS	0.2396	0.2897	0.2524	0.0373	0.76	JS
		SC	0.0686	0.0987	0.0848	0.0139	0.82	SC
		WWS	0.0083	0.0045	0.0065	–0.0020	**0.58**	TSE
	JS	SC	0.1857	0.1883	0.1882	0.0001	**0.62**	SC
		WWS	–0.4141	–0.4329	–0.4183	–0.0146	1	JS
	SC	WWS	0.0004	0.0068	0.0875	–0.0807	0.72	SC

*P(cau), P(cause). Boldface entries are the values of P(cause) smaller than 0.7.*

**TABLE 10:** Summary of GMC for Turkey.

Code	Scenario		*ρ*	GMC(X| Y)	GMC(Y| X)	*δ*	P(cau)	Kernel cause
	X	Y						
TUR	TSE	JS	0.3609	0.3636	0.3802	–0.0166	1	TSE
		SC	0.1173	0.1329	0.1951	–0.0622	1	TSE
		WWS	–0.1466	–0.1546	–0.1985	0.0439	1	WWS
	JS	SC	0.2481	0.2610	0.2890	–0.0280	0.94	JS
		WWS	–0.4285	–0.4349	–0.4317	–0.0032	1	JS
	SC	WWS	–0.0292	–0.1004	–0.1216	0.0212	**0.6**	WWS

*P(cau), P(cause). Boldface entries are the values of P(cause) smaller than 0.7.*

**TABLE 11:** Summary of GMC for Vietnam.

Code	Scenario		*ρ*	GMC(X| Y)	GMC(Y| X)	*δ*	P(cau)	Kernel cause
	X	Y						
VNM	TSE	JS	0.2535	0.2636	0.2785	–0.0149	0.9	TSE
		SC	0.1469	0.1997	0.1911	0.0086	1	SC
		WWS	–0.0205	–0.1134	–0.1289	0.0155	1	WWS
	JS	SC	0.2110	0.2743	0.2860	–0.0117	0.94	JS
		WWS	–0.1564	–0.2237	–0.1721	–0.0516	**0.5**	JS
	SC	WWS	–0.0157	–0.0861	–0.1057	–0.0196	0.92	SC

*P(cau), P(cause). Boldface entries are the values of P(cause) smaller than 0.7.*

**FIGURE 6:**
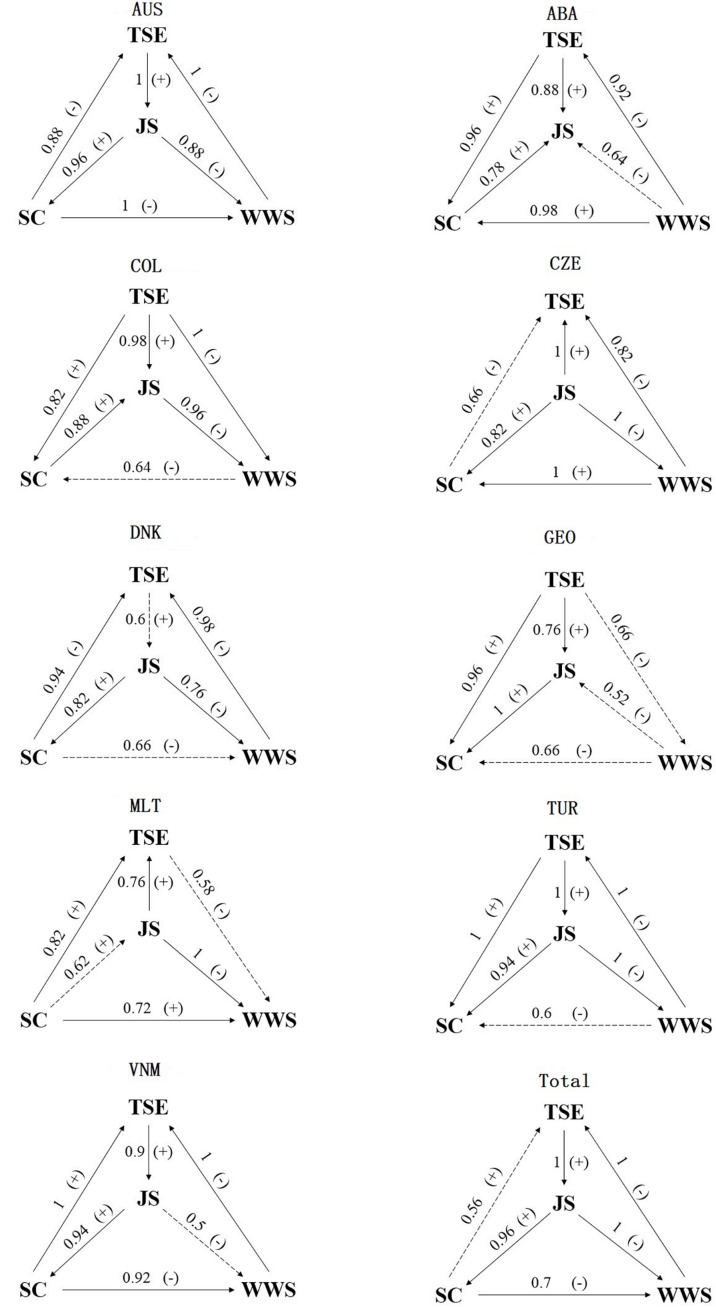
Kernel causal paths of the nine countries/economics and total samples. P(cause) is marked on the line. “+” denotes positive correlated, and “–” denotes negative correlated. The solid line means kernel causality with acceptable probability, and the dotted line means unconvinced kernel causality.

Comparing the pair of GMC, we can obtain the kernel cause. The probability of kernel causality is presented as P(cause) in the 8*th* column of Tables 3–11. Figure 6 summarizes the kernel causal paths for different countries/economics and total samples, which presents the kernel causality graphically. The solid line means kernel causality with acceptable probability [i.e., P(cause)≥ 0.7], and the dotted line means unconvinced kernel causality [i.e., P(cause) < 0.7].

No kernel causality is always acceptable for all countries/economies. Comparing these countries/economies, most unconvinced kernel causalities appear when considering the relationship between WWS and other factors. For Australia, 3 of 6 kernel causal directions are identified with probability 1; 1 of 6, 3 of 6, 1 of 6, 1 of 6, 4 of 6, and 2 of 6 kernel causal directions are determined with probability 1 for Columbia, the Czechia, Georgia, Malta, Turkey, and Vietnam, respectively. Only for Australia, all kernel causalities are acceptable with P(cause) larger than 0.88. As a result, the kernel causality for Turkey is the most stable, even though the probability of kernel causal direction from WWS to SC is 0.6. And the kernel causality among these factors for Georgia is the least stable with 3 unconvinced kernel causal directions.

As a reference, Table 12 presents the GMC and corresponding information for total samples. For the total samples, the kernel causal direction from SC to TSE is unconvinced with probability 0.56. The directions for Australia, Denmark, and Vietnam are the same as those for total samples, but with different probabilities. Among these nine countries/economics and total samples, an explicit kernel causal factor^5^ exists in five countries/economics: WWS for CBAB – Argentina, TSE for Colombia, JS for the Czechia, TSE for Georgia, and SC for Malta. Different kernel cause may result from different histories, cultures, and educational ecologies. On the other hand, a preferable kernel causal factor^6^ is recommended in four other countries/economics and total samples.

**TABLE 12:** Summary of GMC for total samples.

Scenario		*ρ*	GMC(X| Y)	GMC(Y| X)	*δ*	P(cau)	Kernel cause
X	Y						
TSE	JS	0.3309	0.3326	0.3468	–0.0142	1	TSE
	SC	0.0705	0.1256	0.1168	0.0088	**0.56**	SC
	WWS	–0.0742	–0.0896	–0.1411	0.0515	1	WWS
JS	SC	0.2095	0.2196	0.2293	–0.0097	0.96	JS
	WWS	–0.3557	–0.3657	–0.3620	–0.0037	1	JS
SC	WWS	–0.0427	–0.0909	–0.0792	–0.0117	0.7	SC

*P(cau), P(cause). Boldface entries are the values of P(cause) smaller than 0.7.*

## Discussion

In the present study, teacher self-efficacy, job satisfaction, school climate, and workplace well-being and stress are shown to be related to teacher turnover (Borg, 1990; Warr, 1999; Tschannen-Moran and Hoy, 2001; Wang et al., 2015). Clarifying the relation among such factors can help researchers deeply explore the development path of high teaching quality and help policy makers formulate more efficient educational policy systems. In this article, we analyzed the TALIS-PISA linked data with extreme response style using the nominal response model with non-normal latent trait assumption. We compared them with multi-group analysis and explored the kernel causalities of each country/economy.

Comparison results of LTs are slightly different from those of total scores, and the non-normal LT assumption is found to be reasonable. In terms of Pearson correlation coefficients and GMC, WWS is almost negatively correlated with other factors, and most other correlated relations are positive. Kernel cause is obtained by comparing the pair of GMC. Regarding the kernel causality, all causal directions for Australia are acceptable, of which the rejection probabilities of H_0_ are all larger than the cut-off point, but for other countries/economies, at least one causal direction is unconvinced (i.e., at least one rejection probability is smaller than the cut-off point). On the other hand, different explicit or preferable kernel causal factors are identified for different countries/economics, that may result from different histories, cultures, and educational ecologies.

### Educational Implications

These four factors analyzed in this article have influences on teacher’s willingness of turnover. In the field of scientific research, this study adds to a growing body of research on relation among teacher’s traits and educational large-scale survey (e.g., TALIS) analysis. The findings can inform teacher development and educational decision making.

To promote the professional and psychological development of teachers, some potentially effective interventions directed in an explicit or preferable kernel causal factor should be assessed, such as comprehensive training to help teachers develop a sense of self-efficiency (Burić and Moe, 2020; Fackler et al., 2021), a positive workplace-based program to promote teachers’ job satisfaction (Ansley et al., 2019), or a customized mindfulness-based program to reduce teachers’ stress and increase their wellbeing (Beshai et al., 2016).

For education policy makers, this study provides a novel direction to make policies for teachers with different purposes, as the trait may be changed by moderating its cause. It is worth noting that our results suggest that heterogeneous cultures and local characteristics lead to different kernel causal paths. Therefore, when making educational decision, strategies should be adjusted across different countries/economics.

### Limitations

To the best of our knowledge, this is the first study to assess kernel causality of teachers’ traits (i.e., self-efficacy, job satisfaction, school climate, and workplace well-being and stress in this article) using GMC. While the results are informative, this study can be extended in a number of directions. First, the mediation effects of these factors can be evaluated and compared. Second, as the TALIS-PISA linked data are available, the relation among such factors can also be investigated from the perspective of principals and/or students. The conclusion obtained through different angles of view will be more objective with more credibility. The relation between teaching quality and students’ achievement or students’ well-being can also be examined. In addition, we can adopt network analysis to understand how teachers’ similarity and dissimilarity impact the willingness to turnover. Third, the influences of other factors (e.g., burnout, distributed leadership, or emotion regulation) should be considered and compared to seek an effective mechanism for teachers’ psychological health (Rew, 2013; Liu and Hallinger, 2018; Moè and Katz,2020a,b). Furthermore, we can use the multilevel or/and multidimensional nominal response model to analyze ERS (Ju and Falk, 2019). Finally, international comparative study of educational policies and education ecological environment should be conducted to explain the different causal paths among such factors.

## Conclusion

The assessment of kernel causality indicates the explicitly preferable kernel causal factor among these four factors. Further, the results of the multi-group analysis discussed in this article support the hypothesis that there exist extreme response styles and the rationale to adopt non-normal assumption of latent traits’ distribution. These findings contribute to the literature on quantitative research of causality, beyond the existing knowledge based on correlation or association. In addition, our study has identified important new areas to be considered when exploring the relationship among teachers’ and students’ traits under educational settings.

## Data Availability Statement

Publicly available datasets were analyzed in this study. This data can be found here: https://www.oecd.org/education/talis/talis-2018-data.htm OECD, TALIS.

## Author Contributions

XZ provided original thoughts and key technical support and completed the writing of the article. CZ did the data analysis. YX reviewed the literature. SL and ZW provided key theoretical support. All authors contributed to the article and approved the submitted version.

## Conflict of Interest

The authors declare that the research was conducted in the absence of any commercial or financial relationships that could be construed as a potential conflict of interest.

## Publisher’s Note

All claims expressed in this article are solely those of the authors and do not necessarily represent those of their affiliated organizations, or those of the publisher, the editors and the reviewers. Any product that may be evaluated in this article, or claim that may be made by its manufacturer, is not guaranteed or endorsed by the publisher.

## Footnotes

1 As this causality depends on the kernel estimators of GMC, which are calculated by a nonparametric method using kernel function, it is named as “*kernel causality*”. As a result, kernel causality can relax the assumption of variables’ distribution.

3 The bootstrap sampling number is set to be 50.

4 The mean, standard deviation, skewness, and excess kurtosis are calculated by IBM SPSS statistics 26.

5 The explicit kernel causal factor is defined as the factor which is always a kernel cause of other factors.

6 The preferable kernel causal factor is defined as the factor which is a kernel cause of more than half the factors (i.e., 2 of 3 factors in this research).

## Appendix

**APPENDIX TABLE 1:** Items used in this study from TALIS 2018.

Scale	Subscale	Items	Original TALIS code
TSE	SECLS	TT3G34D, TT3G34F, TT3G34H, TT3G34I	T3SECLS
	SEINS	TT3G34C, TT3G34J, TT3G34K, TT3G34L	T3SEINS
	SEENG	TT3G34A, TT3G34B, TT3G34E, TT3G34G	T3SEENG
JS	JSENV	TT3G53C*, TT3G53E, TT3G53G, TT3G53J	T3JSENV
	JSPRO	TT3G53A, TT3G53B, TT3G53D*, TT3G53F*	T3JSPRO
	SATAT	TT3G40A, TT3G40B, TT3G40C, TT3G40D, TT3G40E	T3SATAT
SC	DISC	TT3G41A, TT3G41B*, TT3G41C, TT3G41D	T3DISC
	STUD	TT3G49A, TT3G49B, TT3G49C, TT3G49D	T3STUD
	STAKE	TT3G48A, TT3G48B, TT3G48C, TT3G48D, TT3G48E	T3STAKE
WWS	WELS	TT3G51A, TT3G51B*, TT3G51C, TT3G51D	T3WELS
	WLOAD	TT3G52A, TT3G52B, TT3G52E, TT3G52C, TT3G52D	T3WLOAD
	STBEH	TT3G52F, TT3G52G, TT3G52H	T3STBEH

*TSE, teacher self-efficacy; SECLS, self-efficacy in classroom management; SEINS, self-efficacy in instruction; SEENG, self-efficacy in student engagement; JS, job satisfaction; JSENV, job satisfaction with work environment; JSPRO, job satisfaction with profession; SATAT, satisfaction with target class autonomy; SC, school climate; DISC, teachers’ perceived disciplinary climate; STUD, teacher-student relations; STAKE, participation among stakeholders, teachers; WWS, workplace well-being and stress; WELE, workplace well-being and stress;WLOAD, workload stress; STBEH, student behavior stress. *Denotes the reverse coded item.*

**Source*: OECD, TALIS 2018 Database.*

## References

[B1] Ainley J. Carstens R. (2018). *Teaching and Learning International Survey (TALIS) 2018 Conceptual Framework*, OECD Education Working Papers, No. 187. Paris: OECD Publishing. 10.1787/799337c2-en

[B2] Ansley B. M. Houchins D. Varjas K. (2019). Cultivating positive work contexts that promote teacher job satisfaction and retention in high-need schools. *J. Spec. Educ. Lead.* 32, 3–16.

[B3] Armor D. Conroy-Oseguera P. Cox M. King N. McDonnell L. Pascal A. (1976). *An Analysis of the School Preferred Reading Programs in Selected Los Angeles Minority Schools.* REPORT NO. R-2007-LAUSD. Santa Monica, CA: Rand Corporation (ERIC Document Reproduction Service No.130243).

[B4] Bandura A. (1977). Self-efficacy: toward a unifying theory of behavioral change. *Psychol. Rev.* 84:191e215. 10.1037/0033-295X.84.2.191 847061

[B5] Bandura A. (1997). *Self-Efficacy: The Exercise of Control.* New York, NY: W.H. Freeman and Company.

[B6] Bangs J. Frost D. (2012). *Teacher Self-Efficacy, Voice and Leadership: Towards a Policy Framework for Education International.* Cambridge: University of Cambridge Education International Research Institute.

[B7] Baumgartner H. Steenkamp J. B. E. M. (2001). Response styles in marketing research: a cross-national investigation. *J. Mark. Res.* 38 143–156. 10.1509/jmkr.38.2.143.18840 11670861

[B8] Beshai S. McAlpine L. Weare K. Kuyken W. (2016). A non-randomised feasibility trial assessing the efficacy of a mindfulness-based intervention for teachers to reduce stress and improve well-being. *Mindfulness* 7, 198–208. 10.1007/s12671-015-0436-1

[B9] Björnsson J. K. (2020). “Teaching culturally diverse student groups in the nordic countries—what can the TALIS 2018 data tell us?,” in *Equity, Equality and Diversity in the Nordic Model of Education*, eds Frønes T. S. Pettersen A. Radišić J. Buchholtz N. (Cham: Springer), 75–97. 10.1007/978-3-030-61648-9_4

[B10] Bock R. D. (1972). Estimating item parameters and latent ability when responses are scored in two or more nominal categories. *Psychometrika* 37 29–51. 10.1007/bf02291411

[B11] Borg M. G. (1990). Occupational stress in British educational settings: a review. *Educ. Psychol.* 10:103. 10.1080/0144341900100201

[B12] Buckley J. (2009). “Cross-national response styles in international educational assessments: evidence from PISA 2006,” in *NCES Conference on the Program for International Student Assessment: What We Can Learn from PISA.* Available online at: https://edsurveys.rti.org/PISA/documents/Buckley_PISAresponsestyle.pdf (accessed December 14, 2015).

[B13] Burić I. Moe A. (2020). What makes teachers enthusiastic: the interplay of positive affect, self-efficacy and job satisfaction. *Teach. Teach. Educ.* 89:103008. 10.1016/j.tate.2019.103008

[B14] Chalmers R. P. (2012). mirt: a multidimensional item response theory package for the R environment. *J. Stat. Softw.* 48 1–29. 10.18637/jss.v048.i06

[B15] Cohen J. McCabe E. M. Michelli N. M. Pickeral T. (2009). School climate: research, policy, teacher education and practice. *Teach. Coll. Record* 111 180–213.

[B16] Collie R. J. Shapka J. D. Perry N. E. (2012). School climate and social–emotional learning: predicting teacher stress, job satisfaction, and teaching efficacy. *J. Educ. Psychol.* 104:1189. 10.1037/a0029356

[B17] Colligan T. W. Higgins E. M. (2006). Workplace stress: etiology and consequences. *J. Workplace Behav. Health* 21 89–97. 10.1300/j490v21n02_07

[B18] Dicke T. Marsh H. W. Parker P. D. Guo J. Riley P. Waldeyer J. (2020). Job satisfaction of teachers and their principals in relation to climate and student achievement. *J. Educ. Psychol.* 112 1061–1073. 10.1037/edu0000409

[B19] Evans L. (1997). Understanding teacher morale and job satisfaction. *Teach. Teach. Educ.* 13 831–845. 10.1016/s0742-051x(97)00027-9

[B20] Fackler S. Malmberg L. E. Sammons P. (2021). An international perspective on teacher self-efficacy: personal, structural and environmental factors. *Teach. Teach. Educ.* 99:103255. 10.1016/j.tate.2020.103255

[B21] Granziera H. Perera H. N. (2019). Relations among teachers’ self-efficacy beliefs, engagement, and work satisfaction: a social cognitive view. *Contemp. Educ. Psychol.* 58, 75–84. 10.1016/j.cedpsych.2019.02.003

[B22] Harter J. K. Schmidt F. L. Keyes C. L. (2002). “Well-being in the workplace and its relationship to business outcomes: a review of the Gallup studies,” in *Flourishing: The Positive Person and the Good Life*, eds Keyes C. L. Haidt J. (Washington, DC: American Psychological Association), 205–224. 10.1037/10594-009

[B23] Hoy W. K. Woolfolk A. E. (1993). Teachers’ sense of efficacy and the organizational health of schools. *Elem. Sch. J.* 93 355–372. 10.1086/461729

[B24] Hui C. H. Triandis H. C. (1989). Effects of culture and response format on extreme response style. *J. Cross Cult. Psychol.* 20 296–309. 10.1177/0022022189203004

[B25] Ju U. Falk C. F. (2019). Modeling response styles in cross-country self-reports: an application of a multilevel multidimensional Nominal Response Model. *J. Educ. Meas.* 56 169–191. 10.1111/jedm.12205

[B26] Kim I. Loadman W. E. (1994). *Predicting Teacher Job Satisfaction.* Columbus, OH: Ohio State University.

[B27] Lazarus R. S. (1966). *Psychological Stress and the Coping Process.* New York, NY: McGraw-Hill.

[B28] Lévi L. (2000). Stressors at the workplace: theoretical models. *Occup. Med.* 15 69–106. 10620787

[B29] Liu S. Hallinger P. (2018). Principal instructional leadership, teacher self-efficacy, and teacher professional learning in China: testing a mediated-effects model. *Educ. Adm. Q.* 54, 501–528. 10.1177/0013161X18769048

[B30] Locke E. A. (1976). “The nature and causes of job satisfaction,” in *Handbook of Industrial and Organizational Psychology*, ed. Dunnette M. D. (Chicago, IL: Rand McNally), 1297–1343.

[B31] Moè A. Katz I. (2020a). Emotion regulation and need satisfaction shape a motivating teaching style. *Teach. Teach.* 1–18. 10.1080/13540602.2020.1777960

[B32] Moè A. Katz I. (2020b). Self-compassionate teachers are more autonomy supportive and structuring whereas self-derogating teachers are more controlling and chaotic: the mediating role of need satisfaction and burnout. *Teach. Teach. Educ.* 96:103173. 10.1016/j.tate.2020.103173

[B33] OECD (2019). *TALIS 2018 Technical Report.* Paris: OECD Publishing.

[B34] Renzulli L. A. Parrott H. M. Beattie I. R. (2011). Racial mismatch and school type teacher satisfaction and retention in charter and traditional public schools. *Sociol. Educ.* 84 23–48. 10.1177/0038040710392720

[B35] Rew W. J. (2013). *Instructional Leadership Practices and Teacher Efficacy Beliefs: Cross-National Evidence from TALIS.* Unpublished doctoral dissertation. Tallahassee, FL: Florida State University.

[B36] Schwarzer R. Hallum S. (2008). Perceived teacher self-efficacy as a predictor of job stress and burnout: mediation analyses. *Appl. Psychol.* 57 152–171. 10.1111/j.1464-0597.2008.00359.x

[B37] Smylie M. A. (1988). The enhancement function of staff development: organizational and psychological antecedents to individual teacher change. *Am. Educ. Res. J.* 25 1–30. 10.3102/00028312025001001

[B38] Spector P. E. (1997). *Job Satisfaction: Application, Assessment, Causes, and Consequences.* London: Sage.

[B39] Thapa A. Cohen J. Guffey S. Higgins-D’Alessandro A. (2013). A review of school climate research. *Rev. Educ. Res.* 83 357–385. 10.3102/0034654313483907

[B40] Tschannen-Moran M. Hoy A. W. (2001). Teacher efficacy: capturing an elusive construct. *Teach. Teach. Educ.* 17 783–805. 10.1016/s0742-051x(01)00036-1

[B41] Vinod H. D. (2017). Generalized correlation and kernel causality with applications in development economics. *Commun. Stat. Simul. Comput.* 46 4513–4534. 10.1080/03610918.2015.1122048

[B42] Vinod H. D. (2020). *generalCorr: Generalized Correlations and Initial Causal Path: R package version 1.1.8.* New York, NY: Fordham University.

[B43] Wang H. Hall N. C. Rahimi S. (2015). Self-efficacy and causal attributions in teachers: effects on burnout, job satisfaction, illness, and quitting intentions. *Teach. Teach. Educ.* 47 120–130. 10.1016/j.tate.2014.12.005

[B44] Wang K. Chen Z. Luo W. Li Y. Waxman H. (2018). Examining the differences between the job satisfaction of STEM and non-STEM novice teachers with leaving intentions. *EURASIA J. Math. Sci. Technol. Educ.* 14 2329–2341. 10.29333/ejmste/89516

[B45] Warr P. (1990). The measurement of well-being and other aspects of mental health. *J. Occup. Psychol.* 63 193–210. 10.1111/j.2044-8325.1990.tb00521.x

[B46] Warr P. (1999). “Well-being and the workplace,” in *Well-Being: The Foundations of Hedonic Psychology*, eds Kahneman D. Deiner E. Schwarz N. (New York, NY: Russell Sage), 392–412.

[B47] Wilson C. Marks Woolfson L. Durkin K. (2020). School environment and mastery experience as predictors of teachers’ self-efficacy beliefs towards inclusive teaching. *Int. J. Incl. Educ.* 24, 218–234. 10.1080/13603116.2018.1455901

[B48] Woods C. M. (2006). Ramsay-curve item response theory (RC-IRT) to detect and correct for nonnormal latent variables. *Psychol. Methods* 11 253–270. 10.1037/1082-989X.11.3.253 16953704

[B49] Woods C. M. (2007a). Empirical histograms in item response theory with ordinal data. *Educ. Psychol. Meas.* 67 73–87. 10.1177/0013164406288163

[B50] Woods C. M. (2007b). Ramsay curve IRT for Likert-type data. *Appl. Psychol. Meas.* 31 195–212. 10.1177/0146621606291567

[B51] Woods C. M. (2015). “Estimating the latent density in unidimensional IRT to permit non-normality,” in *Handbook of Item Response Theory Modeling: Applications to Typical Performance Assessment*, eds Reise S. P. Revicki D. A. (New York, NY: Routledge), 60–84.

[B52] Woods C. M. Thissen D. (2006). Item response theory with estimation of the latent population distribution using spline-based densities. *Psychometrika* 71:281. 10.1007/s11336-004-1175-8 28197961

[B53] Zheng S. Shi N. Z. Zhang Z. (2012). Generalized measures of correlation for asymmetry, nonlinearity, and beyond. *J. Am. Stat. Assoc.* 107 1239–1252. 10.1080/01621459.2012.710509

[B54] Zullig K. J. Koopman T. M. Patton J. M. Ubbes V. A. (2010). School climate: historical review, instrument development, and school assessment. *J. Psychoeduc. Assess.* 28 139–152. 10.1177/0734282909344205

